# Nephropulmonary fistula with coralliform stone migration into the thorax: a case report

**DOI:** 10.31744/einstein_journal/2024RC1220

**Published:** 2024-11-22

**Authors:** João Marco Braz Scarpa Mariano Pereira, Leonardo de Oliveira Antunes, Laiane Bicho Janegitz, Matheus José Maia Pereira, Luiz Carlos Maciel, Alberto Kalil Kobbaz

**Affiliations:** 1 Universidade de Taubaté Taubaté SP Brazil Universidade de Taubaté, Taubaté, SP, Brazil.; 2 Universidade de Taubaté Hospital Regional do Vale do Paraíba Taubaté SP Brazil Hospital Regional do Vale do Paraíba, Universidade de Taubaté, Taubaté, SP, Brazil.; 3 Universidade de Taubaté Department of Medicine Taubaté SP Brazil Department of Medicine, Universidade de Taubaté, Taubaté, SP, Brazil.

**Keywords:** Nephropulmonary fistula, Fistula, Nephrolithiasis, Lung abscess, Drainage, Kidney, Migration, Coralliform stone

## Abstract

A nephropulmonary fistula is a rare complication of a non-functioning kidney, associated with a history of infection. Medical literature describes it as an adult disease in the pre-antibiotic era, and nowadays, is a rare complication. This study reports the case of a patient with nephrolithiasis who developed a nephropulmonary fistula resulting in the migration of renal coralliform stones to the lung parenchyma. The treatment included posterior mini-thoracotomy with partial costectomy of the 9^th^ right costal arch, pneumonotomy for the lung abscess, stone removal, abscess drainage with a Foley probe, and pleural drainage with a tubular drain.

## INTRODUCTION

Nephropulmonary fistula is a rare complication of a nonfunctioning kidney, typically associated with a history of infection. Progression to pulmonary abscess or empyema in cases of nephrolithiasis is uncommon.^(1)^ Most described cases were in adults from the pre-antibiotic era.^(2,3)^

Communication between the pleural and retroperitoneal cavities occurs through the lumbocostal triangle of the diaphragm, a relatively fragile area that allows infection to spread from the retroperitoneum to the thoracic cavity.^(4,5)^

The clinical presentation ranges from typical urinary symptoms of pyelonephritis or respiratory problems such as dry cough persisting for weeks or months, to acute respiratory arrest with bronchial blockage of mucus.^(2)^

The goal of this study was to report the diagnosis and treatment of the first case of a nephropulmonary fistula with coralliform stone migration into the thorax during the antibiotic era.

The report was based on an analysis of medical records, case progression, laboratory and imaging tests, and surgical photographs. Furthermore, the bibliographic database included previous studies about nephropulmonary fistula available in the PubMed and SciELO databases.

## CASE REPORT

A 48-year-old woman with type II diabetes mellitus and cognitive impairment presented to the Emergency Care Unit with severe continuous lower back pain, cough, and hemoptysis for 2 months. Whole abdominal ultrasonography showed bilateral obstructive nephrolithiasis with marked hydronephrosis, altered renal morphology, and an image suggestive of a nephropulmonary fistula. Subsequently, she was transferred to *Hospital Regional do Vale do Paraíba* (HRVP), where further laboratory and imaging tests were performed. Upon admission, the patient was tachypneic (25 breaths/min) with an oxygen saturation of 96%. Laboratory tests showed a urea of 147mg/dL and creatinine of 7.1mg/dL (Table 1). Urinalysis revealed a pH of 6.5, leukocytes of 700,000 cells/mL, hemoglobin of 320,000 cells/mL, and negative urine farming. Blood gas analysis showed a pH of 7.26, PCO2 52mmHg, PO2 92.5mmHg, HCO3 14.3mmol/L, lactate 10mmol/L, and BE −7.1mmol/L. Based on these results, empirical treatment with ceftriaxone was initiated.

**Table 1:** Renal function

Period	Urea (mg/dL)	Creatinine (mg/dL)
Hospital admission	147	7.1
After double-J catheter insertion	139	6.65
After renal replacement therapy	78	3.91
1^st^ postoperative day	55	2.96
2^nd^ postoperative day	34	2.20
3^rd^ postoperative day	43	3.01
4^th^ postoperative day	21	2.18
5^th^ postoperative day	33	2.94

**Figure 1:**
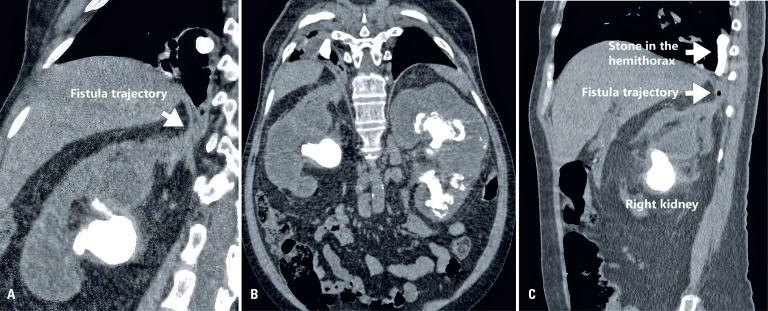
A) Computed tomography (CT) scan of the abdomen shows bilateral coralliform stones with pyelocalycial dilation. B) CT scan of the abdomen shows the fistulous tract and calculus in the right hemithorax. C) Chest CT scan shows calculus in the right kidney, the fistulous tract and the calculus into the right hemithorax

Computed tomography (CT) scan revealed both kidneys were enlarged and had coralliform stones (right stone measuring 5.0 x 4.2cm, density: 1126 HU; largest stone on the left measuring 4.4 x 3.1cm, density: 919 HU, with involvement of all calycinal groups), with obstructions at the ureteropelvic junction of both kidneys (Figure 1A). A fistulous path was observed in the upper pole of the right kidney, resulting in a pulmonary communication (nephropulmonary fistula) and formation of a lung abscess at the right base, with coralliform stones identified above in the diaphragm (in the hemithorax) (Figure 1B). No pneumoperitoneum nor intra- or perirenal gas was observed in this patient; the infectious focus was thus concentrated in the retroperitoneum and ipsilateral thorax.

She underwent bilateral double-J catheter placement but continued to exhibit high levels of nitrogenous waste (Table 1). Therefore, antibiotic therapy was escalated to piperacillin-tazobactam, an Indicate kind of dialysis initiated for clinical stabilization, leading to subsequent improvement in renal function (Table 1).

**Figure 2:**
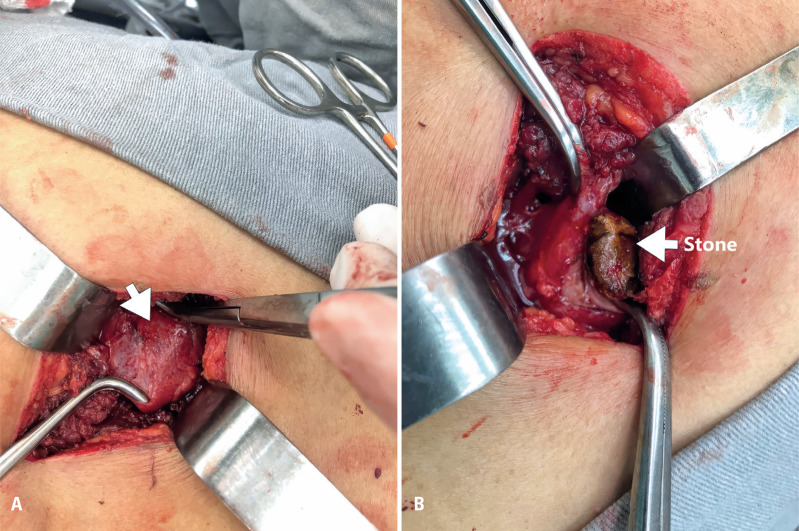
A) Right posterior mini-thoracotomy with lung repair (white arrow) is performed. B) The stones are removed through a pneumonotomy

After multidisciplinary discussion, surgical treatment for the stones in the lung was decided. The patient underwent CT-guided marking of the lung abscess (Figure 1C). Subsequently, a semi-pronated posterior mini-thoracotomy via a 4cm incision (Figure 2A) was performed, with partial costectomy of the 9^th^ right costal arch, pneumonotomy with stone removal (Figure 2B), abscess drainage using a Foley probe, and pleural drainage with a tubular drain.

Postoperatively, the patient showed clinical improvement and a progressive decrease in nitrogenous waste levels (Table 1). The chest tube was removed on the 10^th^ postoperative day, and she was discharged. Unfortunately, the patient progressed to end-stage renal failure, requiring renal replacement therapy through hemodialysis, with follow-up for 1 year at the same institution. She died 1 year postoperatively due to urosepsis.

This study was approved by the Research Ethics Committee of the *Universidade de Taubaté,* CAAE: 76064523.0.0000.5501; # 6.823.580.

## DISCUSSION

A nephropulmonary fistula, although a rare complication of kidney infections, is the second most common complication after nephrocolonic fistulas.^(3)^ It usually occurs secondary to abdominal trauma, complicated lithiasis, or xanthogranulomatous pyelonephritis.^(2,3)^

The association between renal and ipsilateral pulmonary infections in the lower lobe should raise clinical suspicion for a nephropulmonary fistula. Clinical presentation can be nonspecific but usually includes renal symptoms, such as lower back pain, or pulmonary symptoms, such as productive cough, vomiting, and/or uremic breathing.^(4)^ In this case, the patient had cough with hemoptysis, lower back pain, and kidney dysfunction.

Literature reviews indicate the following common characteristics among affected patients: 1) infected kidneys (especially nonfunctional kidneys), obstructive stones, and xanthogranulomatous pyelonephritis; 2) ipsilateral lower lobe pneumonia or pleural effusion; and 3) identical microorganisms in sputum and urine cultures.^(3,6,7)^ The described complications include abscess formation and urinothorax;^(8)^ renal stone migration to the thoracic cavity, however, is unprecedented.

Caberwal et al. reported a patient with a chronic lung abscess 4 years prior to the diagnosis of a nephropulmonary fistula secondary to a pelvic abscess. Imaging of the ipsilateral lung (bronchial infiltrate in the lower lobe, abscess, and effusion) led to the suspicion of this disease^(9)^ highlighting its rarity.

Regarding diagnostic investigations, literature indicates that chest radiography and CT scans may not always show changes suggestive of nephropulmonary fistula or renal gas content.^(1)^ In this case, the diagnosis was made using abdominal CT, which revealed hyperdense imaging (renal calculus) in the pleuropulmonary region permeated by liquid-gaseous content (abscess).

Treatment options vary, but in cases where conservative management, including antibiotics, endourological procedures for stone removal, and percutaneous drainage, is unsuccessful,^(1)^ nephrectomy may be required. In this case, owing to the patient's critical condition, the thoracic fistula was first addressed, and the stone was removed from the pulmonary parenchyma. During surgery, the urology team decided to implant a double-J ureteral stent, with plans for further intervention in a subsequent surgical procedure. Although the affected kidney was not removed during this operation, the patient showed improvement following the removal of the thoracic infectious focus and escalation of antibiotic therapy.

## CONCLUSION

Nephropulmonary fistulas are rare. Patients with the condition may be oligosymptomatic or present with a variety of serious complications, such as lung abscess and urinothorax, making their management challenging. We employed a broad therapeutic approach, addressing both renal and pulmonary involvement, resulting in successful treatment after mini-thoracotomy, stone removal, and lung abscess drainage.
